# Efficacy and safety of CT‐P39, an omalizumab biosimilar, in chronic spontaneous urticaria: 16‐week follow‐up study

**DOI:** 10.1002/clt2.70069

**Published:** 2025-06-02

**Authors:** Clive Grattan, Yevgeniya Dytyatkovska, Michał Springer, Maria Ratkova, Borislava Krusheva, Izabella Krupa‐Borek, Grazyna Pulka, Marta Chełmińska, Adam Reich, Sunghyun Kim, Yunju Bae, Suyoung Kim, Sewon Lee, Eunjin An, Jeong Eun Park, Jieun Ka, Jongho Kim, Sarbjit S. Saini

**Affiliations:** ^1^ St John's Institute of Dermatology Guy's Hospital London UK; ^2^ Clinical Hospital of Emergency Medical Care of Dniprovska City Council Dnipro Ukraine; ^3^ Specjalistyczny NZOZ Alergologia Plus Poznań Poland; ^4^ Medical Center Hera Sofia Bulgaria; ^5^ DCC Alexandrovska Medical University of Sofia Sofia Bulgaria; ^6^ Centrum Alergologii Specjalistyczna Przychodnia Alergologiczna Lublin Poland; ^7^ Centrum Medyczne All‐med Kraków Poland; ^8^ Uniwersyteckie Centrum Kliniczne GUMed Klinika Alergologii Gdańsk Poland; ^9^ Department of Dermatology Institute of Medical Sciences Medical College of Rzeszów University Rzeszów Poland; ^10^ Celltrion, Inc. Incheon Republic of Korea; ^11^ Johns Hopkins Asthma & Allergy Center Baltimore Maryland USA

**Keywords:** chronic spontaneous urticaria, CT‐P39, follow‐up period, omalizumab, treatment switching

## Abstract

**Background:**

A double‐blind, randomized Phase 3 study (NCT04426890) confirmed that CT‐P39 and European Union‐approved reference omalizumab (ref‐OMA) were comparable in terms of efficacy, quality of life (QoL), pharmacokinetics (PK), pharmacodynamics (PD), safety, and immunogenicity up to week 24. Here, we report results from the 16‐week follow‐up period.

**Methods:**

The study included two 12‐week treatment periods (TPs) and a 16‐week off‐treatment follow‐up period. In TP1, 619 patients with chronic spontaneous urticaria (CSU) were randomized to CT‐P39 300 mg, ref‐OMA 300 mg, CT‐P39 150 mg, or ref‐OMA 150 mg. A total of 579 patients continued into TP2, in which patients treated with ref‐OMA 300 mg were rerandomized to CT‐P39 300 mg or to continue on ref‐OMA 300 mg; patients initially randomized to CT‐P39 300 mg continued this regimen; and patients initially randomized to CT‐P39 or ref‐OMA 150 mg increased their dose to 300 mg. Efficacy, PK, PD, QoL, safety, and immunogenicity were assessed during the follow‐up period.

**Results:**

Improvements in efficacy outcomes observed in the TPs gradually decreased during the follow‐up period, but did not return to baseline values. Omalizumab serum concentrations that had increased during treatment subsequently decreased during the follow‐up period. After completing treatment at week 24, total and free immunoglobulin E levels returned toward baseline levels. No clinically meaningful differences in QoL, safety, or immunogenicity outcomes were observed across the treatment groups.

**Conclusion:**

Follow‐up results support the biosimilarity of CT‐P39 and ref‐OMA in terms of efficacy, PK, PD, QoL, safety, and immunogenicity in patients with CSU.

## 1 INTRODUCTION

Omalizumab is a humanized monoclonal antibody drug that works by suppressing mast cell and basophil activation via selective binding to human immunoglobulin E (IgE).^1^, ^2^ This reduces levels of free IgE and downregulates the high‐affinity IgE receptor (FcεRI), leading to mitigation of the allergic cascade.^1^, ^2^, ^3^

Omalizumab was approved in 2014 for the treatment of patients with chronic spontaneous urticaria (CSU) who are symptomatic despite H_1_‐antihistamine treatment,^2^ and its use in this setting is recommended in international guidelines.^4^ Despite its effectiveness, access to omalizumab may be limited by its high cost.^5^ Biosimilar drugs offer significant potential to improve access to biological treatments like omalizumab, as they are typically lower in price than the originator drugs.^6^, ^7^, ^8^, ^9^ Biosimilar drugs, which are assessed through dedicated pathways, must demonstrate no clinically meaningful differences in terms of safety and efficacy in comparison with the reference product.^7^, ^8^, ^9^ The biosimilar landscape has grown and matured since the first product was licensed, and many patients have benefited from increased access as a result.^9^

CT‐P39 (Omlyclo^®^; Celltrion) is the first omalizumab biosimilar to be approved by the European Medicines Agency.^10^, ^11^ A Phase 1 study in healthy volunteers (NCT04018313^12^) demonstrated pharmacokinetic (PK) equivalence of CT‐P39 to reference European Union (EU) and United States omalizumab; a tolerable safety profile was also demonstrated.^1^ A subsequent Phase 3, international, multicenter, double‐blind, randomized, active‐controlled, parallel‐group study, conducted in patients with CSU who had symptoms despite treatment with H_1_‐antihistamines (NCT04426890^13^), demonstrated comparability between CT‐P39 and EU‐approved reference omalizumab (ref‐OMA) in terms of efficacy and safety up to week 24.^14^ For the primary endpoint (mean change from baseline to week 12 in weekly itch severity score [ISS7]), therapeutic equivalence was demonstrated for CT‐P39 300 mg and ref‐OMA 300 mg, as the confidence intervals for the treatment differences were contained within the prespecified equivalence margin.^14^ Secondary efficacy endpoints were also comparable between CT‐P39 and ref‐OMA at both the 300‐mg and 150‐mg dose levels.^14^

Presented here are the efficacy, PK, PD, safety, quality of life (QoL), and immunogenicity results from the 16‐week off‐treatment follow‐up period of the Phase 3 study.

## 2 METHODS

### 2.1 Study design

**FIGURE 1:**
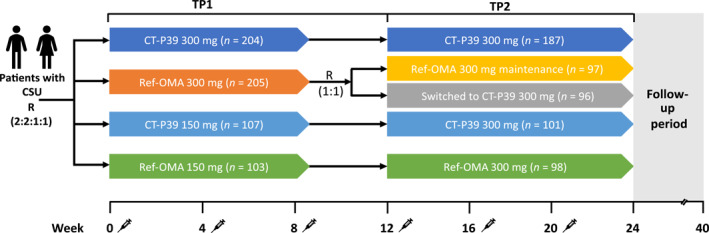
Overview of the study design. CSU, chronic spontaneous urticaria; R, randomized; ref‐OMA, European Union‐approved reference omalizumab; TP, treatment period.

This was a Phase 3, international, multicenter, double‐blind, randomized, active‐controlled, parallel‐group study conducted at 62 study centers in six countries, details of which have been published previously.^14^ The study consisted of a 4‐week screening period followed by two 12‐week treatment periods (TPs) and a 16‐week follow‐up period,^14^ as shown in Figure 1. Overall, 619 patients were randomized in TP1 and 579 patients in TP2. In TP1, patients were randomized (2:2:1:1) to CT‐P39 300 mg (*n* = 204), ref‐OMA 300 mg (*n* = 205), CT‐P39 150 mg (*n* = 107), or ref‐OMA 150 mg (*n* = 103). In TP2, patients initially randomized to ref‐OMA 300 mg were rerandomized (1:1) to switch to CT‐P39 300 mg (*n* = 96) or to continue on ref‐OMA 300 mg (*n* = 97); patients initially randomized to CT‐P39 300 mg continued the same treatment regimen (*n* = 187); and patients initially randomized to CT‐P39 or ref‐OMA 150 mg continued receiving the same drug at an increased dose of 300 mg (CT‐P39 300 mg, *n* = 101; ref‐OMA 300 mg, *n* = 98). Study drugs were administered subcutaneously by prefilled syringe at weeks 0, 4, and 8 (TP1); and weeks 12, 16, and 20 (TP2). All patients continued to receive background medication with one non‐sedating H_1_‐antihistamine at an approved and stable dose throughout the study.^14^ During the follow‐up period, patients could add one additional non‐sedating H_1_‐antihistamine, if required, as a background medication. In addition to background medications, rescue therapy with an H_1_‐antihistamine for itch relief was permitted at a stable dose throughout the study, as required. Thus, up to three H_1_‐antihistamines could be used during the study. The use of steroids and other biological agents targeting IgE was not permitted during the study. After TP2, patients were followed for an additional 16 weeks, with visits scheduled every 4 weeks until the end‐of‐study visit at week 40. Patients were asked to continue to participate in the study assessments even after prematurely discontinuing the study drug.

The study was conducted according to Good Clinical Practice, principles of the Declaration of Helsinki, and applicable local laws or regulations, and was preregistered with EudraCT (2020‐000952‐36)^15^ and ClinicalTrials.gov (NCT04426890).^13^ Study materials were approved by independent ethics committees/institutional review boards at each site and all patients gave written informed consent before starting the study.^14^

### 2.2 Patients

The study included male and female patients aged 12–75 years who had been diagnosed with CSU (refractory to treatment with H_1_‐antihistamines) for ≥6 months. Full details of the inclusion and exclusion criteria for the study have been published previously.^14^

### 2.3 Study endpoints and assessments

Data for efficacy assessments were collected via patient eDiary, a mobile device application installed on the patients' smartphones. Patients were instructed to complete the eDiary twice daily (morning and evening) from screening until the end‐of‐study visit.^16^, ^17^ As with the TP, efficacy assessments during the follow‐up period comprised changes from baseline in: ISS7, weekly hives severity score (HSS7), weekly urticaria activity score (UAS7), and number of antihistamine tablets used per week as rescue therapy. QoL endpoints during the follow‐up period were changes from baseline in overall Dermatology Life Quality Index (DLQI) score and in the overall Chronic Urticaria QoL Questionnaire (CU‐Q_2_oL) score.

Following completion of the TPs, PK (serum concentration of omalizumab) and PD (total and free IgE) continued to be evaluated during the follow‐up period. Safety was assessed throughout the study, including monitoring of treatment‐emergent adverse events (TEAEs), treatment‐emergent serious adverse events (TESAEs), vital signs, electrocardiogram, and laboratory parameters. TEAEs of special interest included allergic reaction type I/anaphylaxis, injection‐site reactions, allergic reaction type III (serum sickness/serum sickness‐like reaction), and parasitic (helminth) infections. Blood samples for immunogenicity assessments were collected at the end of the follow‐up period. Samples were analyzed for levels of antidrug antibodies (ADA) and neutralizing antibodies using a validated immunoassay.

### 2.4 Data analysis

All endpoints were summarized descriptively, and no formal statistical tests were conducted during the follow‐up period. Missing data (ISS7, HSS7, and UAS7) were handled as follows: (i) when either the morning or evening score was missing, the non‐missing morning or evening score was used as the daily score; (ii) if a patient had completed ≥4 eDiary daily scores in a week, the weekly score was calculated as the sum of available scores adjusted for the number of available days; and (iii) if a patient had completed <4 daily scores in a week, then the weekly score was considered missing. All statistical analyses were conducted using SAS 9.4 (SAS Institute Inc.).

Demographic/disease characteristics are presented at baseline. Efficacy, QoL, PK, PD, and immunogenicity results are presented up to week 40. For safety assessments, results are presented for the 16‐week follow‐up period (week 24 to week 40) and the overall study period (week 0 to week 40). Results are presented for the following six groups: (i) CT‐P39 300 mg (all patients randomized to CT‐P39 300 mg in TP1); (ii) ref‐OMA 300 mg (all patients randomized to ref‐OMA 300 mg in TP1); (iii) initial ref‐OMA 300 mg: switched to CT‐P39 300 mg (patients randomized to ref‐OMA 300 mg in TP1 and rerandomized to CT‐P39 300 mg in TP2 [TP2 analysis subset]); (iv) initial ref‐OMA: ref‐OMA 300 mg maintenance (patients randomized to ref‐OMA 300 mg in TP1 and rerandomized to continue ref‐OMA 300 mg in TP2 [TP2 analysis subset]); (v) CT‐P39 150 mg (patients randomized to CT‐P39 150 mg in TP1); and (vi) ref‐OMA 150 mg (patients randomized to ref‐OMA 150 mg in TP1).

As patients could enter the follow‐up period despite discontinuing the study drug, patients who discontinued the study drug during TP1 or TP2 were included in the follow‐up period analysis. For the two groups created following the second randomization of the ref‐OMA 300 mg group (initial ref‐OMA 300 mg switched to CT‐P39 300 mg, and ref‐OMA 300 mg maintenance), only patients who entered into TP2 were included, as designated by “TP2” analysis sets. The various analysis sets are fully defined in the Supporting Information.

## 3 RESULTS

### 3.1 Patient disposition

Of the 619 patients randomized in this study, 554 entered the additional 16‐week follow‐up period (Table S1). Overall, a total of 518 patients completed the study, including the follow‐up period. The proportion of patients who completed the study up to week 40 ranged from 80.6% to 88.7% across the groups. The number of patients who discontinued during TP1 or TP2 along with the reasons for discontinuation are detailed in Table S1. There were no differences in early drug discontinuation rates or reasons for discontinuation between the groups (Table S1). Overall, the most common primary reason for study termination in the follow‐up period was “other” (4 [2.0%], 5 [2.4%], 5 [5.2%], 0, 4 [3.7%], and 3 [2.9%] patients in the CT‐P39 300 mg, ref‐OMA 300 mg, initial ref‐OMA: switched to CT‐P39 300 mg, initial ref‐OMA: ref‐OMA 300 mg maintenance, CT‐P39 150 mg, and ref‐OMA 150 mg groups, respectively). Among them, one patient in the CT‐P39 300 mg group discontinued the study during the follow‐up period due to worsening of CSU. Baseline demographics and disease characteristics were similar across the groups (Table 1). Across the groups, most patients were female (57.7%–69.9%) and White (78.1%–81.4%), and approximately one‐third had angioedema at baseline (25.2%–39.2%).

**TABLE 1:** Demographics, disease characteristics, and stratification factors at baseline (RAN set, with RAN‐TP2 for rerandomized patients).

	CT‐P39 300 mg (*n* = 204)	Ref‐OMA 300 mg^a^ (*n* = 205)	Initial ref‐OMA		CT‐P39 150 mg^b^ (*n* = 107)	Ref‐OMA 150 mg^b^ (*n* = 103)
			Switched to CT‐P39 300 mg (*n* = 96)	Ref‐OMA 300 mg maintenance (*n* = 97)		
Median age (range), years	43 (15–71)	41 (12–75)	41 (13–69)	40 (12–75)	42 (13–75)	42 (13–72)
Female, *n* (%)	133 (65.2)	131 (63.9)	65 (67.7)	56 (57.7)	67 (62.6)	72 (69.9)
Mean height at screening (SD), cm	169.0 (9.1)	167.6 (8.5)	166.8 (8.4)	168.3 (8.6)	169.3 (8.6)	169.1 (9.3)
Mean weight at screening (SD), kg	76.5 (17.1)	76.7 (17.3)	77.0 (18.5)	77.3 (16.4)	75.8 (17.4)	75.6 (17.5)
Mean BMI at screening (SD), kg/m^2^	26.7 (5.3)	27.2 (5.4)	27.6 (5.9)	27.2 (4.8)	26.4 (5.5)	26.4 (5.4)
Race, *n* (%)						
White	166 (81.4)	165 (80.5)	75 (78.1)	78 (80.4)	86 (80.4)	83 (80.6)
Asian	38 (18.6)	40 (19.5)	21 (21.9)	19 (19.6)	21 (19.6)	20 (19.4)
Ethnicity, *n* (%)						
Hispanic or Latino	0	0	0	0	1 (0.9)	0
Non‐Hispanic or Non‐Latino	204 (100.0)	205 (100.0)	96 (100.0)	97 (100.0)	106 (99.1)	103 (100.0)
Angioedema present, *n* (%)	65 (31.9)	70 (34.1)	30 (31.3)	38 (39.2)	27 (25.2)	40 (38.8)
Mean number of antihistamine tablets per week of rescue therapy at baseline (SD)^c^	3.37 (3.07)	3.65 (3.65)	3.30 (3.22)	3.64 (3.17)	3.18 (3.19)	3.61 (3.90)
Mean baseline ISS7 (SD)^c^	15.68 (3.64)	15.27 (3.86)	15.31 (3.63)	15.36 (3.54)	15.53 (3.30)	15.75 (3.26)
Mean baseline UAS7 (SD)^c^	31.74 (7.11)	31.20 (7.49)	31.04 (7.29)	31.70 (7.06)	31.43 (6.95)	32.22 (6.99)
Mean baseline HSS7 (SD)^c^	16.07 (4.56)	15.93 (4.50)	15.73 (4.40)	16.34 (4.32)	15.91 (4.55)	16.47 (4.39)

Abbreviations: BMI, body mass index; HSS7, weekly hives severity score; ISS7, weekly itch severity score; mITT, modified intention‐to‐treat; mITT‐TP2, modified intention‐to‐treat set—treatment period 2; RAN, randomized; RAN‐TP2, randomized set—treatment period 2; ref‐OMA, European Union‐approved reference omalizumab; SD, standard deviation; UAS7, weekly urticaria activity score.

^a^ Rerandomized to switching arm and non‐switching arm at week 12.

^b^ Dose increased from 150 to 300 mg at week 12.

^c^ Data from mITT set (mITT‐TP2 for rerandomized patients): CT‐P39 300 mg (*n* = 203); ref‐OMA 300 mg (*n* = 205); switched to CT‐P39 300 mg (*n* = 96); ref‐OMA 300 mg maintenance (*n* = 96); CT‐P39 150 mg (*n* = 107); ref‐OMA 150 mg (*n* = 103).

### 3.2 Efficacy

**FIGURE 2:**
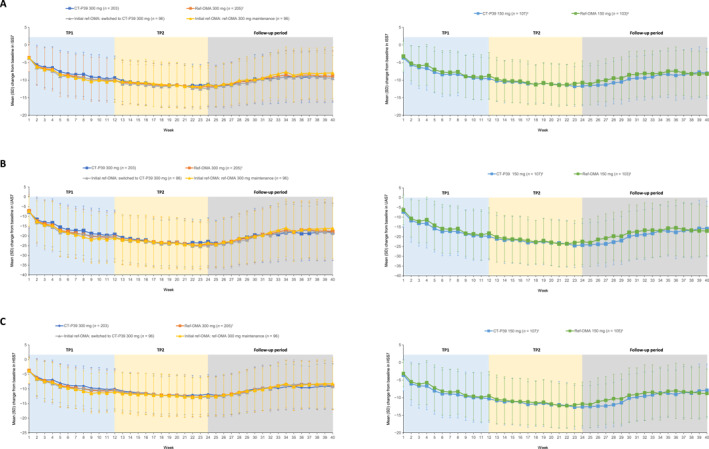
All observed mean (±SD) changes from baseline to the end of the follow‐up period for (A) ISS7, (B) UAS7, and (C) HSS7 (mITT set, with mITT‐TP2 for rerandomized patients). ^†^Rerandomized to switching arm and non‐switching arm at week 12. ^‡^Dose increased from 150 to 300 mg at week 12. HSS7, weekly hives severity score; ISS7, weekly itch severity score; mITT, modified intention‐to‐treat; mITT‐TP2, modified intention‐to‐treat set—treatment period 2; ref‐OMA, European Union‐approved reference omalizumab; SD, standard deviation; TP, treatment period; UAS7, weekly urticaria activity score.

Mean change from baseline in ISS7 was similar across treatment groups at week 40 (end of study; Table 2). After notable improvement in ISS7 was observed after the overall 24‐week TP, improvement in ISS7 score gradually decreased up to week 40 but did not return to baseline mean values, and there were no notable differences among the treatment groups during the follow‐up period (Table 2; Figure 2A). Similar findings were observed for UAS7 (Table 2; Figure 2B) and HSS7 (Table 2; Figure 2C). Changes in the number of antihistamine tablets used as rescue therapy per week were similar across treatment groups. Overall, although some numerical variance between the groups was apparent, the mean number of antihistamine tablets used per week as rescue therapy at week 40 was lower than at baseline in all treatment groups (Table 2). During the follow‐up period, less than 10% of patients in all treatment groups initiated an additional non‐sedating H_1_‐antihistamine as a second background medication (11 [5.4%], 11 [5.4%], 6 [6.3%], 5 [5.2%], 4 [3.7%], and 10 [9.7%] patients in the CT‐P39 300 mg, ref‐OMA 300 mg, initial ref‐OMA: switched to CT‐P39 300 mg, initial ref‐OMA: ref‐OMA 300 mg maintenance, CT‐P39 150 mg, and ref‐OMA 150 mg groups, respectively).

**TABLE 2:** Summary of efficacy results at week 40 (mITT set, with mITT‐TP2 for rerandomized patients).

Mean change from baseline, SD	CT‐P39 300 mg (*n* = 203)	Ref‐OMA 300 mg^a^ (*n* = 205)	Initial ref‐OMA		CT‐P39 150 mg^b^ (*n* = 107)	Ref‐OMA 150 mg^b^ (*n* = 103)
			Switched to CT‐P39 300 mg (*n* = 96)	Ref‐OMA 300 mg maintenance (*n* = 96)		
ISS7	−8.91 (7.39)	−8.89 (6.95)	−9.47 (6.54)	−7.94 (7.26)	−8.02 (7.11)	−8.22 (6.25)
UAS7	−18.07 (14.59)	−17.72 (14.79)	−18.59 (13.96)	−16.11 (15.51)	−15.91 (14.10)	−17.03 (12.65)
HSS7	−9.16 (7.67)	−8.83 (8.28)	−9.12 (7.81)	−8.18 (8.77)	−7.89 (7.44)	−8.81 (6.94)
Number of antihistamine tablets per week of rescue therapy	−0.31 (5.16)	−1.20 (5.13)	−1.57 (4.60)	−0.71 (5.68)	−0.22 (4.77)	−1.50 (4.77)
DLQI score^c^	−6.6 (8.4)	−6.8 (9.0)	−7.0 (9.8)	−6.6 (8.5)	−6.9 (8.9)	−6.2 (7.8)
CU‐Q_2_oL score^c^	−20.00 (23.46)	−19.11 (26.97)	−19.74 (27.73)	−19.94 (25.72)	−18.82 (23.66)	−20.07 (24.31)

Abbreviations: CU‐Q_2_oL, Chronic Urticaria Quality of Life Questionnaire; DLQI, Dermatology Life Quality Index; HSS7, weekly hives severity score; ISS7, weekly itch severity score; mITT, modified intention‐to‐treat; mITT‐TP2, modified intention‐to‐treat set—treatment period 2; ref‐OMA, European Union‐approved reference omalizumab; SD, standard deviation; UAS7, weekly urticaria activity score.

^a^ Rerandomized to switching arm and non‐switching arm at week 12.

^b^ Dose increased from 150 to 300 mg at week 12.

^c^ Week 40/end of study.

After notable improvement after the overall 24‐week TP,^14^ improvement in DLQI and CU‐Q_2_oL scores decreased but did not return to baseline at the end of the follow‐up period, and there were no notable differences between treatment groups (Table 2).

### 3.3 Pharmacokinetics

**FIGURE 3:**
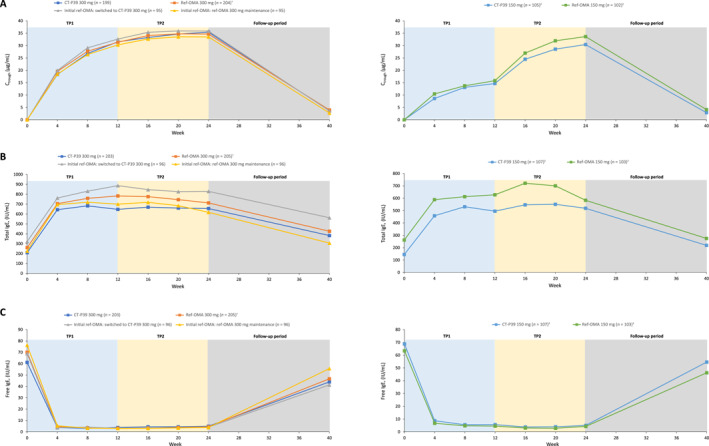
All observed mean serum concentrations of (A) PK (PK set, with PK‐TP2 for rerandomized patients), (B) total IgE, and (C) free IgE (safety set, with safety‐TP2 for rerandomized patients). ^†^Rerandomized to switching arm and non‐switching arm at week 12. ^‡^Dose increased from 150 to 300 mg at week 12. C_trough_, trough serum concentration; IgE, immunoglobulin E; PK, pharmacokinetic; PK‐TP2, pharmacokinetic set—treatment period 2; ref‐OMA, European Union‐approved reference omalizumab; TP, treatment period.

The mean omalizumab trough serum concentration (C_trough_) increased in line with the administered dose during the TPs, and then gradually decreased during the follow‐up period, with no notable differences between the treatment groups (Figure 3A). The mean (standard deviation) serum concentrations of omalizumab at week 40/end of study were 3.67 (6.73), 3.96 (6.49), 3.59 (4.72), 2.74 (4.01), 2.87 (6.10), and 4.11 (7.20) μg/mL in the CT‐P39 300 mg, ref‐OMA 300 mg, initial ref‐OMA: switched to CT‐P39 300 mg, initial ref‐OMA: ref‐OMA 300 mg maintenance, CT‐P39 150 mg, and ref‐OMA 150 mg groups, respectively.

### 3.4 Pharmacodynamics

Mean total IgE levels increased approximately two‐ to three‐fold following study drug administration during the TPs, and then gradually decreased during the follow‐up period in all treatment groups (Figure 3B). Free IgE levels decreased after study drug administration, and gradually recovered toward baseline during the follow‐up period in all treatment groups (Figure 3C).

### 3.5 Safety

During the follow‐up period (week 24 to week 40), the proportions of patients who experienced ≥1 TEAE were similar across treatment groups (Table 3A). The majority of TEAEs were considered unrelated to the study drug. The most frequently reported TEAEs (≥2% patients in any group) were coronavirus disease 2019 (COVID‐19), headache, nasopharyngitis, tonsillitis, and upper respiratory tract infection. Rates of TESAEs ranged from 0% to 3.1% across the treatment groups. All TESAEs were considered unrelated to the study drug. No TEAEs led to death or study drug discontinuation, and no TEAEs were classified as allergic reaction type I/anaphylaxis, injection‐site reactions, serum sickness/serum sickness‐like reaction, or parasitic (helminth) infections.

**TABLE 3:** Summary of TEAEs. (A) Follow‐up period and (B) overall study period (safety set, with safety‐TP2 for rerandomized patients).

Patients, *n* (%)	CT‐P39 300 mg (*n* = 203)	Ref‐OMA 300 mg^a^ (*n* = 205)	Initial ref‐OMA		CT‐P39 150 mg^b^ (*n* = 107)	Ref‐OMA 150 mg^b^ (*n* = 103)
			Switched to CT‐P39 300 mg (*n* = 96)	Ref‐OMA 300 mg maintenance (*n* = 96)		
A						
≥1 TEAE	37 (18.2)	39 (19.0)	19 (19.8)	19 (19.8)	24 (22.4)	20 (19.4)
Related	1 (0.5)	0	0	0	0	1 (1.0)
TEAEs reported by ≥2% patients in any group, *n* (%)						
COVID‐19	2 (1.0)	8 (3.9)	5 (5.2)	3 (3.1)	4 (3.7)	5 (4.9)
Headache	2 (1.0)	0	0	0	3 (2.8)	1 (1.0)
Nasopharyngitis	3 (1.5)	7 (3.4)	4 (4.2)	2 (2.1)	1 (0.9)	1 (1.0)
Tonsillitis	0	2 (1.0)	2 (2.1)	0	0	0
Upper respiratory tract infection	1 (0.5)	2 (1.0)	0	2 (2.1)	0	0
≥1 TESAE	1 (0.5)	4 (2.0)	3 (3.1)	1 (1.0)	3 (2.8)	0
Related	0	0	0	0	0	0
≥TEAE leading to study drug discontinuation	0	0	0	0	0	0
≥1 TEAE leading to death	0	0	0	0	0	0
≥1 TEAE classified as allergic reaction type I/anaphylaxis	0	0	0	0	0	0
≥1 TEAE classified as injection‐site reaction	0	0	0	0	0	0
≥1 TEAE classified as serum sickness/serum sickness‐like reactions	0	0	0	0	0	0
≥1 TEAE classified as parasitic (helminth) infections	0	0	0	0	0	0
B						
≥1 TEAE	89 (43.8)	99 (48.3)	48 (50.0)	47 (49.0)	53 (49.5)	50 (48.5)
Related	17 (8.4)	22 (10.7)	12 (12.5)	9 (9.4)	7 (6.5)	14 (13.6)
TEAEs reported by ≥5% patients in any group, *n* (%)						
COVID‐19	12 (5.9)	18 (8.8)	9 (9.4)	9 (9.4)	10 (9.3)	10 (9.7)
Headache	8 (3.9)	6 (2.9)	1 (1.0)	5 (5.2)	3 (2.8)	6 (5.8)
Injection‐site reaction	5 (2.5)	12 (5.9)	6 (6.3)	5 (5.2)	2 (1.9)	3 (2.9)
Nasopharyngitis	11 (5.4)	14 (6.8)	5 (5.2)	8 (8.3)	9 (8.4)	3 (2.9)
≥1 TESAE	9 (4.4)	6 (2.9)	3 (3.1)	2 (2.1)	4 (3.7)	3 (2.9)
Related	3 (1.5)	0	0	0	1 (0.9)	0
≥TEAE leading to study drug discontinuation	4 (2.0)	2 (1.0)	0	0	0	0
≥1 TEAE leading to death	1 (0.5)^c^	0	0	0	0	0
≥1 TEAE classified as allergic reaction type I/anaphylaxis	0	1 (0.5)	0	0	0	0
≥1 TEAE classified as injection‐site reaction	5 (2.5)	12 (5.9)	6 (6.3)	5 (5.2)	2 (1.9)	3 (2.9)
≥1 TEAE classified as serum sickness/serum sickness‐like reactions	2 (1.0)	0	0	0	0	0
≥1 TEAE classified as parasitic (helminth) infections	0	0	0	0	0	0

Abbreviations: COVID‐19, coronavirus disease 2019; ref‐OMA, European Union‐approved reference omalizumab; TEAE, treatment‐emergent adverse event; TESAE, treatment‐emergent serious adverse event; TP, treatment period.

^a^ Rerandomized to switching arm and non‐switching arm at week 12.

^b^ Dose increased from 150 to 300 mg at week 12.

^c^ Due to suicide.

During the overall study period, the proportions of patients who experienced ≥1 TEAE and ≥1 related TEAE were similar across treatment groups (Table 3B). The majority of TEAEs were considered unrelated to the study drug. Injection‐site reactions were the most commonly reported related TEAEs; however, all injection‐site reactions occurred during the TPs.

The most frequently reported TEAEs (≥5% patients in any group) were COVID‐19, headache, injection‐site reaction, and nasopharyngitis. One patient died due to completed suicide, as previously reported.^14^

There were no notable differences between treatment groups in mean change from baseline for all clinical chemistry, hematology, and urinalysis laboratory parameters during the study. The majority of patients had normal results for physical examination and electrocardiogram assessments at the week 40/end‐of‐study visit.

### 3.6 Immunogenicity

Most patients had negative ADA results during the follow‐up period. The number of patients with positive ADA results at the week 40/end‐of‐study visit was 14 (6.9%), 3 (1.5%), 3 (3.1%), 0, 7 (6.5%), and 2 (1.9%) in the CT‐P39 300 mg, ref‐OMA 300 mg, initial ref‐OMA: switched CT‐P39 300 mg, initial ref‐OMA: ref‐OMA 300 mg maintenance, CT‐P39 150 mg, and ref‐OMA 150 mg groups, respectively.

## 4 DISCUSSION

Our findings demonstrated that, during the whole study and the follow‐up period, CT‐P39 was well tolerated, had a safety profile similar to that of ref‐OMA at each dose level, and resulted in a similar pattern of efficacy. There was a large improvement in disease control during treatment, followed by enduring efficacy (with some decline) after treatment cessation. The efficacy results are in line with historical data from studies with omalizumab that demonstrated a gradual return of symptoms during follow‐up which, as with the current study, did not quite return to the levels seen at baseline.^3^, ^18^ During the 16‐week follow‐up period of this Phase 3 study, similar outcomes were observed for patients who had continued ref‐OMA and those who had switched from ref‐OMA to CT‐P39 at week 12, indicating that efficacy, PK, PD, safety, QoL, and immunogenicity were not impacted by the treatment switch. The difference in total IgE levels after administration observed between CT‐P39 and ref‐OMA is likely attributable to differences in baseline values. Apart from this, following study drug administration, total IgE levels increased two‐ to three‐fold from baseline in both treatment groups, consistent with historical data expectations.^19^, ^20^, ^21^ The overall safety profile of CT‐P39 continues to be consistent with the known safety profile of omalizumab,^3^, ^18^, ^22^, ^23^, ^24^ with no new or unexpected concerns during the whole study period and no new safety findings, including after switching to CT‐P39 from ref‐OMA.

Overall, the immunogenicity outcomes in this study were slightly higher than the known clinical profile with ref‐OMA, which has shown a low risk of immunogenicity in clinical trials and real‐world settings.^25^ The higher ADA incidences reported in this study are likely due to the use of a more sensitive ADA assay compared with the original ref‐OMA studies.^24^ Although CT‐P39 showed numerically higher rates of ADA positivity at week 40 compared with ref‐OMA, the overall incidences were generally low and there was no discernible clinical impact among ADA‐positive patients in relation to efficacy response, PK, and safety. The incidence of TEAEs in ADA‐positive patients was in line with the overall treatment arms. One ADA‐positive patient in the CT‐P39 treatment arm experienced serum sickness/serum sickness‐like reaction that developed before the onset of ADA‐positivity. There were no reports of allergic reaction type 1/anaphylaxis or injection‐site reactions among the ADA‐positive patients. Our data therefore confirm that the immunogenicity risks of CT‐P39 are not clinically different from those of ref‐OMA.

The strengths of this study include its multicenter, international, randomized, double‐blind, and active‐controlled design, plus the wide range of endpoints evaluated, including meticulously and continuously assessed changes in CSU symptoms over time. The limitations previously reported for the primary outcome remain applicable here: the trial was of a short duration, the population was relatively small, and there was a lack of racial diversity among trial participants. Longer‐term studies in larger, more diverse populations are needed to confirm these findings and to identify factors that might impact treatment response.

### 4.1 Conclusions

The omalizumab biosimilar CT‐P39 was previously shown to have equivalent efficacy and comparable PK, PD, safety, and immunogenicity versus ref‐OMA after 24 weeks of treatment, or after switching from ref‐OMA to CT‐P39 at week 12, in patients with CSU who had symptoms despite treatment with H_1_‐antihistamines.^14^ Here, we present the results of the 16‐week follow‐up period. The current analysis supports the equivalence of CT‐P39 to ref‐OMA by demonstrating that the efficacy, PK, PD, safety, and QoL outcomes of patients treated with CT‐P39 were similar to those of patients treated with ref‐OMA up to week 40, including 16 weeks of follow‐up after treatment was completed. Our findings provide further confirmatory evidence that CT‐P39 is a validated biosimilar for omalizumab.

## AUTHOR CONTRIBUTIONS

**Clive Grattan**: Conceptualization; writing—original draft preparation; writing—review and editing. **Yevgeniya Dytyatkovska**: Investigation; writing—original draft preparation; writing—review and editing. **Michał Springer**: Investigation; writing—original draft preparation; writing—review and editing. **Maria Ratkova**: Investigation; writing—original draft preparation; writing—review and editing. **Borislava Krusheva**: Investigation; writing—original draft preparation; writing—review and editing. **Izabella Krupa‐Borek**: Investigation; writing—original draft preparation; writing—review and editing. **Grazyna Pulka**: Investigation; writing—original draft preparation; writing—review and editing. **Marta Chełmińska**: Investigation; writing—original draft preparation; writing—review and editing. **Adam Reich**: Investigation; writing—original draft preparation; writing—review and editing. **Sunghyun Kim**: Conceptualization; methodology; supervision; writing—original draft preparation; writing—review and editing. **Yunju Bae**: Conceptualization; methodology; writing—original draft preparation; writing—review and editing. **Suyoung Kim**: Conceptualization; methodology; writing—original draft preparation; writing—review and editing. **Sewon Lee**: Conceptualization; methodology; writing—original draft preparation; writing—review and editing. **Eunjin An**: Conceptualization; methodology; writing—original draft preparation; writing—review and editing. **Jeong Eun Park**: Conceptualization; methodology; writing—original draft preparation; writing—review and editing. **Jieun Ka**: Formal analysis; methodology; software; writing—original draft preparation; writing—review and editing. **Jongho Kim**: Formal analysis; methodology; software; writing—original draft preparation; writing—review and editing. **Sarbjit S. Saini**: Conceptualization; writing—original draft preparation; writing—review and editing.

## CONFLICT OF INTEREST STATEMENT

**Clive Grattan** has served as a consultant or advisory board member for argenx, Blueprint Medicines, Celltrion, Novartis, Thermo Fisher, and Sanofi; sits on steering committees for AB Science (without payment) and Blueprint Medicines; and has previously chaired a Data Safety Monitoring Board for CSL Behring. **Adam Reich** is or recently was a speaker and/or advisor for, and/or has received research funding from Almirall, Alumis, Alvotech, Amgen, AnaptysBio, argenx, AstraZeneca, Biogen, Boehringer Ingelheim, Bristol Myers Squibb, Celltrion, Chema Rzeszów, Galderma, Horizon, Incyte, InflaRx, Janssen, Kiniksa, Leo Pharma, Lilly, Novartis, Numab, Pfizer, Sanofi/Regeneron, Takeda, Trevi Therapeutics, and UCB. **Sunghyun Kim**, **Yunju Bae**, **Suyoung Kim**, **Sewon Lee**, **Eunjin An**, **Jeong Eun Park**, **Jieun Ka**, and **Jongho Kim** are employees of the study sponsor Celltrion, Inc. **Sarbit S. Saini** has received grant, research, and/or clinical trial support from Allakos, Amgen, Escient, Jasper, National Institutes of Health, Novartis, Regeneron, and Sanofi; and has served as a consultant or advisory board member for Allakos, Aquestive, Celltrion, Escient, Granular Therapeutics, Innate, Novartis, Regeneron, and Sanofi. **Yevgeniya Dytyatkovska**, **Michał Springer**, **Maria Ratkova**, **Borislava Krusheva**, **Izabella Krupa‐Borek**, **Grazyna Pulka**, and **Marta Chełmińska** declare no conflict of interest in relation to this work.

## Supporting information

Supporting Information S1

## Data Availability

The data that support the findings of this study are available in the Supporting Information accompanying this article.

## References

[1] Maurer M , Saini SS , McLendon K , et al. Pharmacokinetic equivalence of CT‐P39 and reference omalizumab in healthy individuals: a randomised, double‐blind, parallel‐group, phase 1 trial. Clin Transl Allergy. 2022;12(11):e12204. 10.1002/clt2.12204 36434739 PMC9665328

[2] Kaplan AP , Giménez‐Arnau AM , Saini SS . Mechanisms of action that contribute to efficacy of omalizumab in chronic spontaneous urticaria. Allergy. 2017;72(4):519‐533. 10.1111/all.13083 27861988 PMC5915348

[3] Maurer M , Rosén K , Hsieh HJ , et al. Omalizumab for the treatment of chronic idiopathic or spontaneous urticaria. N Engl J Med. 2013;368(10):924‐935. 10.1056/nejmoa1215372 23432142

[4] Zuberbier T , Abdul Latiff AH , Abuzakouk M , et al. The international EAACI/GA^2^LEN/EuroGuiDerm/APAAACI guideline for the definition, classification, diagnosis, and management of urticaria. Allergy. 2022;77(3):734‐766. 10.1111/all.15090 34536239

[5] Matsubara D , Takahagi S , Saito R , Kamegashira A , Tanaka A , Hide M . Analysis of the long‐term economic burden of omalizumab on patients with chronic spontaneous urticaria. J Dermatol. 2021;48(1):56‐63. 10.1111/1346-8138.15630 33029864

[6] Kvien TK , Patel K , Strand V . The cost savings of biosimilars can help increase patient access and lift the financial burden of health care systems. Semin Arthritis Rheum. 2022;52:151939. 10.1016/j.semarthrit.2021.11.009 35027243

[7] Constantin MM , Cristea CM , Taranu T , et al. Biosimilars in dermatology: the wind of change. Exp Ther Med. 2019;18(2):911‐915. 10.3892/etm.2019.7505 31384323 PMC6639911

[8] Kim H , Alten R , Avedano L , et al. The future of biosimilars: maximizing benefits across immune‐mediated inflammatory diseases. Drugs. 2020;80(2):99‐113. 10.1007/s40265-020-01256-5 32002851 PMC7007415

[9] Gherghescu I , Delgado‐Charro MB . The biosimilar landscape: an overview of regulatory approvals by the EMA and FDA. Pharmaceutics. 2020;13(1):48. 10.3390/pharmaceutics13010048 33396369 PMC7824407

[10] Celltrion, Inc . Celltrion receives European Commission approval of Omlyclo^®^ (CT‐P39), the first and only omalizumab biosimilar approved in Europe. [press release]. Accessed May 28, 2025. https://celltrion.com/en‐us/company/media‐center/press‐release/3246

[11] European Medicines Agency . Omlyclo summary of product characteristics. Accessed May 28, 2025. https://www.ema.europa.eu/en/documents/product‐information/omlyclo‐epar‐product‐information_en.pdf

[12] ClinicalTrials.gov . NCT04018313. To compare the PK and safety of omalizumab (CT‐P39, EU‐approved Xolair, and US‐licensed Xolair) in healthy subjects. Accessed May 28, 2025. https://clinicaltrials.gov/study/NCT04018313

[13] ClinicalTrials.gov . NCT04426890. To compare efficacy and safety of CT‐P39 and EU‐approved Xolair in patients with chronic spontaneous urticaria (omalizumab). Accessed May 28, 2025. https://clinicaltrials.gov/study/NCT04426890

[14] Saini SS , Maurer M , Dytyatkovska Y , et al. CT‐P39 compared with reference omalizumab in chronic spontaneous urticaria: results from a double‐blind, randomized, active controlled, phase 3 study. Allergy. Epub ahead of print January 9, 2025. 10.1111/all.16446 PMC12368742 39785096

[15] EU Clinical Trials Register . EudraCT number 2020‐000952‐36. A double‐blind, randomized, active‐controlled, parallel group, phase 3 study to compare efficacy and safety of CT‐P39 and Xolair in patients with chronic spontaneous urticaria who remain symptomatic despite H1‐antihistamine treatment. Accessed May 28, 2025. https://www.clinicaltrialsregister.eu/ctr‐search/search?query=2020‐000952‐36

[16] Mathias SD , Dreskin SC , Kaplan A , Saini SS , Spector S , Rosén KE . Development of a daily diary for patients with chronic idiopathic urticaria. Ann Allergy Asthma Immunol. 2010;105(2):142‐148. 10.1016/j.anai.2010.06.011 20674825

[17] Hollis K , Proctor C , McBride D , et al. Comparison of urticaria activity score over 7 days (UAS7) values obtained from once‐daily and twice‐daily versions: results from the ASSURE‐CSU study. Am J Clin Dermatol. 2018;19(2):267‐274. 10.1007/s40257-017-0331-8 29368043 PMC5978890

[18] Saini SS , Bindslev‐Jensen C , Maurer M , et al. Efficacy and safety of omalizumab in patients with chronic idiopathic/spontaneous urticaria who remain symptomatic on H1 antihistamines: a randomized, placebo‐controlled study. J Invest Dermatol. 2015;135(3):67‐75. 10.1038/jid.2014.512 25046337 PMC4269803

[19] Çildağ S , Şentürk T . The effect of omalizumab treatment on IgE and other immunoglobulin levels in patients with chronic spontaneous urticaria and its association with treatment response. Postepy Dermatol Alergol. 2018;35(5):516‐519. 10.5114/ada.2017.71422 30429712 PMC6232555

[20] Mizuma H , Tanaka A , Uchida Y , et al. Influence of omalizumab on allergen‐specific IgE in patients with adult asthma. Int Arch Allergy Immunol. 2015;168(3):165‐172. 10.1159/000442668 26790100

[21] Metz M , Staubach P , Bauer A , et al. Clinical efficacy of omalizumab in chronic spontaneous urticaria is associated with a reduction of FcεRI‐positive cells in the skin. Theranostics. 2017;7(5):1266‐1276. 10.7150/thno.18304 28435464 PMC5399592

[22] European Medicines Agency . Xolair summary of product characteristics. Accessed May 28, 2025. https://www.ema.europa.eu/en/documents/product‐information/xolair‐epar‐product‐information_en.pdf

[23] Kaplan A , Ledford D , Ashby M , et al. Omalizumab in patients with symptomatic chronic idiopathic/spontaneous urticaria despite standard combination therapy. J Allergy Clin Immunol. 2013;132(1):101‐109. 10.1016/j.jaci.2013.05.013 23810097

[24] U.S. Food and Drug Administration . Xolair highlights of prescribing information. Accessed May 28, 2025. https://www.accessdata.fda.gov/drugsatfda_docs/label/2024/103976s5245lbl.pdf

[25] Chen ML , Nopsopon T , Akenroye A . Incidence of anti‐drug antibodies to monoclonal antibodies in asthma: a systematic review and meta‐analysis. J Allergy Clin Immunol Pract. 2023;11(5):1475‐1484.e20. 10.1016/j.jaip.2022.12.046 36716995 PMC10601343

