# Supplementary material for: Efficacy and safety of CT‐P39, an omalizumab biosimilar, in chronic spontaneous urticaria: 16‐week follow‐up study
Source: Clin Transl Allergy. 2025 Jun 2;15(6):e70069. doi: 10.1002/clt2.70069 (PMC12128672; doi:10.1002/clt2.70069)
Supplement: Supplementary file 1 — Supporting Information S1 [file CLT2-15-e70069-s001.docx]

# Supporting information

# Efficacy and safety of CT-P39, an omalizumab biosimilar, in chronic spontaneous urticaria: 16-week follow-up study

**Clive Grattan^1^, Yevgeniya Dytyatkovska^2^, Michal Springer^3^, Maria Ratkova^4^, Borislava Krusheva^5^, Izabella Krupa-Borek^6^, Grazyna Pulka^7^, Marta Chełmińska^8^, Adam Reich^9^, Sunghyun Kim^10^, Yunju Bae^10^, Suyoung Kim^10^, Sewon Lee^10^, Eunjin An^10^, Jeong Eun Park^10^, Jieun Ka^10^, Jongho Kim^10^, Sarbjit S. Saini^11^**

^1^St John’s Institute of Dermatology, Guy's Hospital, London, UK

^2^Clinical Hospital of Emergency Medical Care of Dniprovska City Council, Dnipro, Ukraine

^3^Specjalistyczny NZOZ Alergologia Plus, Poznań, Poland

^4^Medical Center Hera, Sofia, Bulgaria

^5^DCC Alexandrovska Medical University of Sofia, Sofia, Bulgaria

^6^Centrum Alergologii Specjalistyczna Przychodnia Alergologiczna, Lublin, Poland

^7^Centrum Medyczne All-med, Kraków, Poland

^8^Uniwersyteckie Centrum Kliniczne GUMed, Klinika Alergologii, Gdańsk, Poland

^9^Department of Dermatology, Institute of Medical Sciences, Medical College of Rzeszów University, Rzeszów, Poland

^10^Celltrion, Inc., Incheon, Republic of Korea

^11^Johns Hopkins Asthma & Allergy Center, Baltimore, Maryland, USA

## Analysis sets

The randomized (RAN) set included all randomly assigned patients prior to dosing on day 1, regardless of whether they received any study drug; the randomized set – treatment period 2 (RAN-TP2) subset comprised all patients in the RAN set who underwent the second randomization regardless of whether they received either of the study drugs during TP2.

The modified intention-to-treat (mITT) set included all randomly assigned patients who received at least one full dose of either of the study drugs during TP1; the modified intention-to-treat set – TP2 (mITT-TP2) subset comprised all patients in the mITT set who underwent the second randomization and received at least one full dose of either of the study drugs during TP2.

The pharmacokinetic (PK) set included all randomly assigned patients who received at least one full dose of either of the study drugs during TP1 and had at least one post-treatment PK result prior to dosing at week 12. The PK set – TP2 (PK‑TP2) subset comprised all patients in the PK set who received at least one full dose of either of the study drugs during TP2 and had at least one post‑treatment PK result after week 12.

The safety set included all randomly assigned patients who received at least one dose (full or partial) of either of the study drugs; the safety set – TP2 (safety-TP2) subset comprised all patients in the safety set who received at least one dose (full or partial) of either of the study drugs during TP2.

The CT-P39 300 mg, ref-OMA 300 mg, CT-P39 150 mg, and ref-OMA 150 mg groups were all based on the mITT, PK, and safety analysis sets. The initial
ref-OMA 300 mg: switched to CT-P39 300 mg group and initial ref-OMA 300 mg: ref-OMA 300 mg maintenance group were based on the mITT-TP2, PK-TP2, and safety-TP2 sets.

## TABLE S1 Patient disposition during the whole study period (TP1: RAN set; TP2: RAN-TP2 set; follow-up: RAN and RAN-TP2 sets).

| Patients, *n* (%) | CT-P39  300 mg  (*n* = 204) | Ref-OMA  300 mg^†^ (*n* = 205) | CT-P39  150 mg^‡^ (*n* = 107) | Ref-OMA  150 mg^‡^  (*n* = 103) |
| --- | --- | --- | --- | --- |
| Randomized in TP1 | 204 | 205 | 107 | 103 |
| Administered study drug | 203 (99.5) | 205 (100) | 107 (100) | 103 (100) |
| Discontinued study drug | 16 (7.8) | 13 (6.3) | 6 (5.6) | 5 (4.9) |
| Terminated the study in TP1 | 15 (7.4) | 10 (4.9) | 5 (4.7) | 5 (4.9) |
| Primary reason for study drug discontinuation in TP1 | | | | |
| Consent withdrawal | 7 (3.4) | 6 (2.9) | 4 (3.7) | 5 (4.9) |
| Patient decision^§^ | 1 (0.5) | 1 (0.5) | 1 (0.9) | 0 |
| Disease progression | 1 (0.5) | 1 (0.5) | 0 | 0 |
| Lost to follow-up | 0 | 0 | 1 (0.9) | 0 |
| Adverse event | 3 (1.5) | 2 (1.0) | 0 | 0 |
| Significant protocol deviation | 0 | 3 (1.5) | 0 | 0 |
| Other | 4 (2.0) | 0 | 0 | 0 |

| Patients, *n* (%) | CT-P39  300 mg  (*n* = 187) | | Initial ref-OMA | | CT-P39  150 mg^‡^ (*n* = 101) | Ref-OMA  150 mg^‡^  (*n* = 98) | |
| --- | --- | --- | --- | --- | --- | --- | --- |
|  |  |  | **Switched to  CT-P39 300 mg  (*n* = 96)** | **Ref-OMA 300 mg maintenance  (*n* = 97)** |  |  |  |
| Randomized in TP2 | 187 | | 96 | 97 | 101 | 98 | |
| Administered study drug, | 187 (100) | | 96 (100) | 96 (99.0) | 101 (100) | 98 (100) | |
| Discontinued study drug, | 6 (3.2) | | 2 (2.1) | 2 (2.1) | 2 (2.0) | 4 (4.1) | |
| Terminated the study in TP2 | 7 (3.7) | | 6 (6.3) | 5 (5.2) | 4 (4.0) | 8 (8.2) | |
| Primary reason for study drug discontinuation in TP2 | | | | | | | |
| Consent withdrawal | 3 (1.6) | | 1 (1.0) | 0 | 1 (1.0) | 2 (2.0) | |
| Disease progression | 1 (0.5) | | 0 | 0 | 0 | 0 | |
| Lost to follow-up | 0 | | 0 | 1 (1.0) | 0 | 0 | |
| Adverse event | 1 (0.5) | | 0 | 0 | 0 | 0 | |
| Investigator decision | 0 | | 0 | 1 (1.0) | 0 | 1 (1.0) | |
| Other | 1 (0.5) | | 1 (1.0) | 0 | 1 (1.0) | 1 (1.0) | |
| Patients, *n* (%) | **CT-P39  300 mg  (*n* = 204)** | **Ref-OMA  300 mg^†^ (*n* = 205)** | | **Initial ref-OMA** | | **CT-P39  150 mg**^‡^ **(*n* = 107)** | **Ref-OMA  150 mg**^‡^  **(*n* = 103)** |
|  |  |  |  | **Switched to  CT-P39 300 mg (*n* = 96)** | **Ref-OMA  300 mg maintenance (*n* = 97)** |  |  |
| Initiated the follow-up period | 182 (89.2) | 184 (89.8) | | 90 (93.8) | 92 (94.8) | 98 (91.6) | 90 (87.4) |
| Terminated the study in the follow‑up period | 10 (4.9) | 12 (5.9) | | 6 (6.3) | 6 (6.2) | 7 (6.5) | 7(6.8) |
| Completed the study including the follow-up period | 172 (84.3) | 172 (83.9) | | 84 (87.5) | 86 (88.7) | 91 (85.0) | 83 (80.6) |
| Primary reason for study termination in the follow-up period | | | | | | | |
| Consent withdrawal | 4 (2.0) | 4 (2.0) | | 0 | 4 (4.1) | 2 (1.9) | 4 (3.9) |
| Lost to follow-up | 0 | 1 (0.5) | | 0 | 1 (1.0) | 1 (0.9) | 0 |
| Adverse event | 0 | 0 | | 0 | 0 | 0 | 0 |
| Investigator’s decision | 2 (1.0) | 2 (1.0) | | 1 (1.0) | 1 (1.0) | 0 | 0 |
| Study terminated by sponsor | 0 | 0 | | 0 | 0 | 0 | 0 |
| Other | 4 (2.0) | 5 (2.4) | | 5 (5.2) | 0 | 4 (3.7) | 3 (2.9) |

^†^Rerandomized to switching arm and non-switching arm at week 12.
^‡^Dose increased from 150 mg to 300 mg at week 12. ^§^Patients continued to attend regular study visits.
Abbreviations: RAN, randomized; RAN-TP2, randomized set – treatment period 2; ref‑OMA, European Union‑approved reference omalizumab; TP, treatment period.
